# An Overview of the Purity Characteristics, Pigments, and Tocopherol Contents of 48 Virgin Olive Oils from Apulian Minor Olive Accessions

**DOI:** 10.3390/foods14060964

**Published:** 2025-03-12

**Authors:** Giacomo Squeo, Pamela Laera, Roccangelo Silletti, Carmine Summo, Francesco Caponio

**Affiliations:** Food Science and Technology Unit, Department of Soil Plant and Food Sciences, University of Bari “Aldo Moro”, Via Amendola 165/A, 70126 Bari, Italy; p.laera9@phd.uniba.it (P.L.); roccangelo.silletti@uniba.it (R.S.); carmine.summo@uniba.it (C.S.); francesco.caponio@uniba.it (F.C.)

**Keywords:** fatty acids, sterols, genuineness, clusters, antioxidants, biodiversity

## Abstract

Together with the most diffused olive cultivars used for virgin olive oil (VOO) extraction, Apulia boasts a plethora of other minor varieties and accessions whose oil characteristics is little or no studied at all. The main novelty of this study is then providing new insights about selected characteristics of 48 VOOs extracted from underexplored Apulian olive accessions. VOOs were extracted at laboratory scale and characterized for their fatty acid and sterol composition, pigments, and total tocopherol contents. Moreover, multivariate approaches were used to study VOOs similarity based on purity criteria. The results show that some VOOs had percentages of specific fatty acids or sterols out of current international limits. This was already observed in previous studies and should be taken into account by regulatory bodies and policy makers. The multivariate analysis of the dataset by PCA and k-means clustering allow to identify the VOO from San Benedetto variety as the most peculiar and, on the whole, a total of five different clusters of samples. Pigments and tocopherols ranges are wide among the oils, highlighting possible candidates for further valorization.

## 1. Introduction

Virgin olive oil (VOO) is one of the pillars of the Mediterranean diet, which is considered one of the most healthy dietary models and, since 2013, has been inscribed in the Representative List of the Intangible Cultural Heritage of Humanity [1]. VOO is the olive oil extracted only by mechanical or other physical means [2,3] that could be classified, depending on its chemical characteristics, in extra virgin, virgin or lampante [3]. Such a classification is based on quality criteria (i.e., free acidity, peroxide value, UV indices, organoleptic assessment, ethyl esters) that are primarily related to the characteristics of the raw material and the manufacturing practices. Purity criteria, on the other hand, are crucial for assessing olive oil’s genuineness and the absence of any fraud, such as the application of prohibited technological practices, the use of additives, or forbidden coadjuvants, the mixture with refined olive oil or other vegetable oils [4]. Among them, fatty acids and sterols play a pivotal role. These parameters are used to unveil fraudulent practices, principally the illegal addition of vegetable oils with different botanical origin to genuine VOO that, unfortunately, are still frequently carried out given the higher commercial value of VOO respect to other vegetable oils [5]. As a matter of example, in Italy, the last report issued by the Ispettorato centrale della tutela della qualità e della repressione frodi dei prodotti agroalimentari (i.e., Central inspectorate for quality protection and fraud prevention in the food sector) revealed that the practice of pass off seed oil, colored with chlorophylls and carotenoids, for extra virgin olive oil, is still an actual threat [6].

Purity criteria can be used for the intended purpose because depend on the plant species [5,7]. However, remarkable variability in fatty acid and sterol composition can be observed depending on other factors, the genotype and the environmental conditions being some of the most important.

Considering the former, for example, it was reported that differences up to 57% in the content of oleic acid are ascribable to the variety [8].

With regard to the second aspect, the environmental/climatic conditions, it is well known that lower temperatures are correlated with higher contents of oleic acid and with lower contents of palmitic and/or linoleic acids [8]. Indeed, the fact that peculiar environmental contexts can strongly alter the fatty acid composition, bringing genuine VOO out of legal specifications, has been known for a long time [8,9]. However, currently, concerns about the effect of climatic conditions on the purity features of VOOs are newly exploded due to the climate change issue. Recently, Sevim et al. [10] studied the effect of climate conditions on the purity characteristics of Turkish olive oils, finding critical deviation in delta-7-stigmastenol content.

The composition in fatty acids and sterols affects some quality aspects of VOOs as well. For example, the concentration of oleic and linoleic acids, is directly related to the stability of the product [11,12,13]. Nevertheless, in the stability—and more in general, in the quality of VOOs—other components are involved, e.g., hydrophilic and lipophilic antioxidants and pigments [13]. The latter, represented by tocopherols, chlorophylls, and carotenoids, have received less attention compared to their hydrophilic counterparts.

The composition in fatty acids, sterols, tocopherols and pigments of VOOs can vary widely and has multifaceted practical implications, highlighting how this still represents a hot topic of research. This is particularly relevant considering that despite the renewed attention and interest towards olive biodiversity, in the main producing countries only a small fraction of the whole available olive varieties are used for VOO production. In Italy, about 80 cultivars out of 538 accounted for 90% of the total cultivated area [14]. This means that there is a large pool of unexplored, understudied and underknown minor olive varieties or accessions, and derived minor VOOs, worthy to study and characterize. Providing insights about their features goes in the direction of possible valorization strategies and revitalization of rural and marginal areas. Indeed, the strong product–territory linkage, as observed in geographical designations, can have positive impacts on the economy of those territories [15].

On these premises, this work aims primarily at presenting the purity characteristics, tocopherols and pigments of a pool of 48 VOOs extracted from Apulian minor olive accessions. Those have been little or never studied before, representing the main novelty of the current research. Moreover, their similarities/differences based on the fatty acids and sterols composition were studied using multivariate approaches.

## 2. Materials and Methods

A total of 48 olive accessions were provided (about 2 kg), after being hand-harvested at their usual maturity degree from marginal areas of the Apulian provinces already reported in a previous work [16] (Supplementary Table S1) and subjected to VOO extraction using a small pilot plant [17]. The extraction system was made up of a semi-industrial scale hammer crusher (RETSCH GmbH 5657, Haan, Germania) provided with three hammers and a grid with 5 holes on the bottom to let the olive paste fall down. The angular velocity was set at 2850 rpm. The obtained olive paste was indirectly heated at 30 ± 1 °C by means of hot water and mixed for 15 min. Thereafter, the oily phase was recovered by means of a basket centrifuge (Marelli Motori S.p.A., Arzignano, VI, Italia). Once extracted, the oils were stored in 100 mL dark glass bottles until the analyses. The fatty acid profile was determined as reported in [18]. Briefly, samples were mixed with 1 mL of hexane (Honeywell International, Inc., Morristown, NJ, USA) and underwent transesterification with KOH 2N in methanol (Honeywell International, Inc., Morristown, NJ, USA). An aliquot (1 μL) of the upper layer was injected into a GC system (Agilent 7890A gas chromatograph, Agilent Technologies, Santa Clara, CA, USA) equipped with an FID detector (set at 220 °C) and an SP2340 capillary column (60 m × 0.25 mm i.d. × 0.2 mm film thickness; Supelco Park, Bellefonte, PA, USA). The identification of each fatty acid was carried out by comparing the retention time with that of the corresponding standard methyl ester (Sigma-Aldrich, St. Louis, MO, USA). The amount of single fatty acids was expressed as area percentage with respect to the total area.

For sterol composition, about 5 g of oil was weighted, added with α-colestanol (Sigma-Aldrich, St. Louis, MO, USA) as an internal standard, and subjected to saponification with a solution (2 N) of KOH in ethanol (VWR BDH Chemicals, Rou d’Aurion, France) under heating. After that, the sample was washed three times with ethyl ether in a separating funnel to collect the unsaponifiable fraction. The etheric phase was neutralized and filtered by sodium sulphate anhydrous and successively brought to dryness. The sterol fraction, resuspended (5%) in chloroform (Carlo Erba Reagents S.r.l., Cornaredo, Italy), was separated from the unsaponifiable matter by tin layer chromatography and then recovered, filtered, and silanized. Finally, about 1 μL of the solution was injected in the GC system using a capillary column HP-5 30 m × 0.32 mm i.d. × 0.25 μm film thickness (Agilent Technologies, Santa Clara, CA, USA). The injector temperature was 290 °C with a split ratio of 1:25. The carrier gas was helium at a flow rate of 1.3 mL/min. Single sterol content was reported as area percentage with respect to the total sterols area, while the total content was calculated using the internal standard method and expressed as mg/kg [19].

Total carotenoids and chlorophylls were determined spectrophotometrically [20,21]. After filtration, an aliquot of sample was dissolved in hexane and the absorbance at 445 nm registered by using a Cary 60 UV–Vis spectrophotometer (Agilent Technologies, Santa Clara, CA, USA). Carotenoid content was calculated by an external calibration curve of b-carotene (Sigma-Aldrich, St. Louis, MO, USA) and the results expressed as mg of b-carotene per kg of oil. For chlorophyll determination, the absorbances at 630 nm, 670 nm, and 710 nm were measured against an empty cuvette as blank. The values of absorbance were used for calculation as reported in [21] and the results expressed as mg of pheophytin a per kg of oil. Tocopherols were determined by RP-UHPLC-FLD (Dionex Ultimate 3000 RSLC, Waltham, MA, USA) as previously reported [22]. Briefly, an aliquot of oil was dissolved in iso-propanol HPLC grade (Honeywell International, Inc., Morristown, NJ, USA), vortexed and filtered. Then, 20 μL were injected into the HPLC system using fluorescence detection (excitation at 295 nm, emission at 325 nm). The separation was achieved by an isocratic elution of 1:1 (*v*/*v*) acetonitrile/methanol at a flow rate of 1 mL/min. A C18 column was used as stationary phase (Acclaim 120, 150 × 3 mm i.d., particle size 3 μm, ThermoScientific, Waltham, MA, USA). Quantitation was achieved by the external calibration method. Alpha, beta, and gamma isoforms were then summed and the results reported as total tocopherols in mg/kg of oil.

Chemical analyses were carried out in duplicate on one batch of VOO per accession and the mean value presented. The relative standard deviation was always lower than 10%. The descriptive statistics of the dataset were calculated in Microsoft Excel (Microsoft Corporation, Redmond, WA, USA). Principal component analysis (PCA) was carried out on the autoscaled data matrix considering the purity characteristics. Clustering was carried out using the PCA scores as input, and the k-means algorithm. The best number of clusters, between 1 to 10, was defined by running the algorithm 1000 times and using the silhouette index [23,24]. Multivariate analyses were carried out using Matlab (2024b, The MathWorks, MA, USA) and the PLS_toolbox (Eigenvector Research Inc., Manson, WA, USA).

## 3. Results and Discussion

The fatty acid composition, and the relative descriptive statistics, is reported in Table 1. Oleic acid was the most abundant fatty acid, from about 61% to about 81%, with a range in the order of 20%. Palmitic acid was the second most abundant fatty acid, followed by linoleic, stearic acid, and palmitoleic acids. As can be proven by the close agreement between the mean value and the median value, no extreme samples or outliers were present. In general, the fatty acid composition reflects that typical of olive oils. The range observed for the main fatty acids is in line with the variability observed in other studies [25,26]. However, it should be noted that some VOOs did not match the current limit set by IOC [2]. In particular, some samples (highlighted in bold) showed higher concentrations of myristic acid (6/48), palmitoleic acid (1/48), arachidic acid (20/48), and behenic acid (2/48). These results are in line with previous reports that show how, depending on different factors (e.g., variety, crop season, edaphoclimatic conditions), modifications in the fatty acid profile can place genuine VOOs out of the legal specifications [14,27]. Such conclusions can be extended to some of the oils considered in this study, highlighting the need for further studies. Nonetheless, policy makers and regulation bodies should take this issue into account in the context of olive biodiversity valorization.

Sterols are biosynthesized in the olive tree, and their concentrations are sensitive to climatic conditions, variety, degree of ripening, year of harvest, and geographical origin [28]. Table 2 shows the sterol composition of the VOOs under examination together with the descriptive statistics. The close agreement between the mean value and the median value confirm that no extreme samples or outliers were present in the dataset under study. However, also in this case, few oils did not fulfill the legal requirements for some parameters (highlighted in bold), as was the case for brassicasterol (6/48), stigmastenol (2/48), and apparent beta-sitosterol (9/48). Two samples presented values of delta-7-stigmastenol higher that 0.5 but respect the ratio of apparent beta-sitosterol /campesterol ≥ 28, as reported by IOC [2]. Sevim et al. [10] already observed value higher than the limit in several cultivars in different harvest years, concluding that it might be due to variety characteristics as well as to high temperatures. Other reports have demonstrated that sterols composition could deviate from the approved range in genuine oils [29,30,31,32], posing the urgency of further investigating this topic.

Concerning the total sterol content, all the cultivars exceed 1000 mg/kg, in compliance with current legislation. It is worth noting that the range was very wide, although the variability was in line with that observed by [29], considering 43 cultivars from the World Olive Germplasm Bank of Cordoba. The oils studied were extracted from different accessions that also came from different locations within the Apulia region. Hence, ascribing the observed differences to one factor (i.e., variety), the other (i.e., environment), or a combination of both, is challenging, and the experimental plan followed in this study did not allow us to do so. Further studies can be designed to properly deepen this topic.

**Figure 1:**
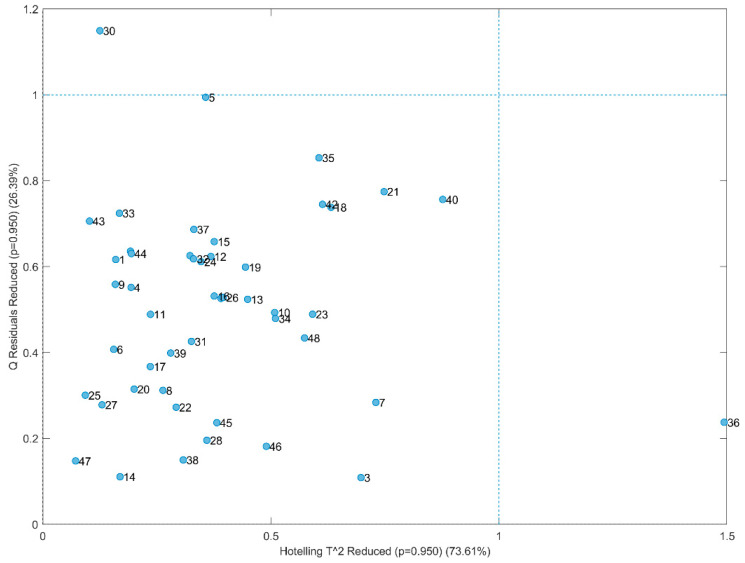
Influence plot.

**Figure 2:**
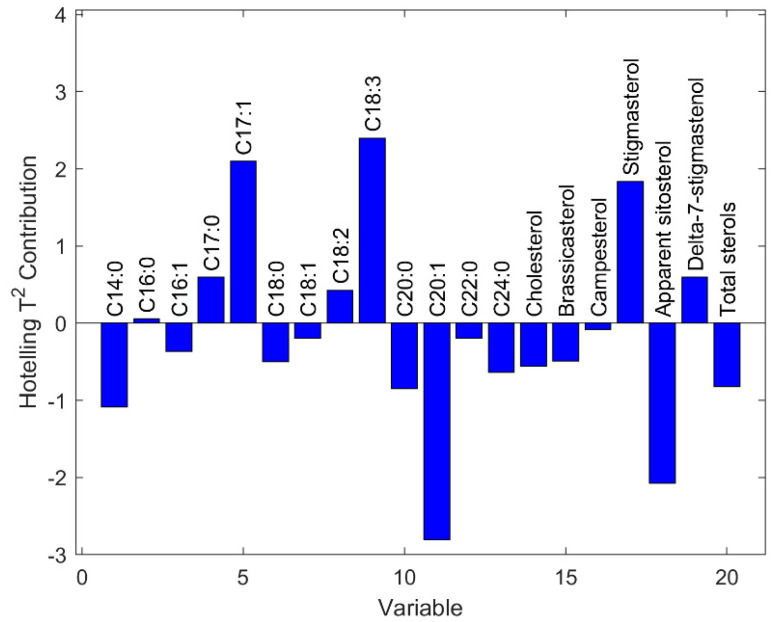
Hotelling’s T^2^ contribution plot for the San Benedetto cultivar.

The purity characteristics of the studied VOOs were used as input for unsupervised multivariate analyses aimed at studying possible similarities/dissimilarity between them and extract further information from the dataset. Firstly, a PCA was carried out. Figure 1 depicts the so-called influence plot, which presents the *Q*-statistics (i.e., residuals) vs. Hotelling’s T^2^ [33]. Samples having high *Q* have characteristics that do not follow the general data structure modelled by the PCA. On the other hand, Hotelling’s T^2^ indicates samples that are within the model—i.e., they share the same variable structure or pattern with the other samples—but to some extent are far from the average. The plot revealed that one sample (no. 36, San Benedetto) presented high value of the Hotelling’s T^2^ statistics. The relative contribution plot depicted in Figure 2 shows that this behavior was linked to some variables, namely C17:1, C18:3, C20:1, stigmasterol and apparent beta-sitosterol. A close inspection of Table 1 and Table 2 in fact allows to appreciate that San Benedetto VOO was characterized by the highest percentage of heptadecenoic acid, linolenic acid, and stigmasterol and by the lowest percentage of gadoleic acid and apparent beta-sitosterol. Some information about this variety has been found in the OLEADB database [34]. The variety is reported to be autochthonous from Italy but not only from Apulia region. In fact, some records indicate the variety as also native from Sicily and Sardinia, where it is called with other synonyms like Termitana or Sanbenedettese.

PCA has the advantage of summarizing the data information into a reduced number of principal components, which can be used in the place of the original variables to study the similarity between samples [23]. Hence, PCA scores have been used for a k-means clustering. The optimal number of clusters according to the silhouette parameter was 5. Table 3 indicates the samples belonging to each identified cluster together with the average characteristics per each of them.

The largest cluster was #3, followed by #4 and #5. It is noteworthy that the least numerous was Cluster #2, which was composed of only one variety—San Benedetto, which was previously highlighted for its peculiar characteristics. Samples from Cluster #1 were, on average, characterized by the lowest amounts of miristic and palmitic acids and the highest amounts of heptadecanoic, gadoleic, and lignoceric acids and brassicasterol. Cluster #2 corresponds to the VOO from San Benedetto variety, whose peculiar characteristics have been already highlighted. Moving to Cluster #3, palmitic, palmitoleic, and linoleic acids were those presented in the highest percentages, while oleic and linolenic acids were presented in the lowest percentages. The sterol composition was intermediate between the other clusters. VOOs belonging to Cluster #4 showed the highest amount of oleic acid and miristic acid on average. On the other hand, these had the lowest percentages of behenic acid. As per the sterol composition, these had the highest amounts of total sterols and campesterol. Finally, VOOs belonging to Cluster #5 were characterized by the highest percentages of stearic, arachidic, and behenic acids while presenting the lowest percentages of heptadecenoic and linoleic acids. Considering the sterols, they had the highest amount of apparent beta-sitosterol and the lowest amount of stigmasterol and delta-7-stigmasterol.

Olive biodiversity is a fundamental tool for the resilience of the olive oil sector. In fact, apart from the common major cultivars usually cultivated for oil production [14], minor varieties and accessions could be the source of additional or diverse characteristics and might represent a leverage for the sector [16]. Historically, one of the gross classifications based on the fatty acid composition of VOOs was that between high-oleic/low-palmitic–linoleic oils and vice versa [27,35]. In our study, samples from Cluster #3 could be recognized as low oleic/high palmitic-linoleic while those from clusters #4 and #5 can be recognized as high oleic/low palmitic-linoleic oils. Nonetheless, it is clear that this classification did not allow us to summarize the complex features of all the samples. This consideration is particularly relevant for minor varieties or accessions that have been little studied and that are potentially able to express a wide range of different features. Hence, the report about the purity characteristics of these minor varieties is welcome and could serve as the basis for their valorization. Moreover, their peculiar characteristics, if confirmed by additional studies, could pose the basis for the identification of authenticity markers for minor varieties.

Finally, Table 4 reports the content of pigments and tocopherols together with the descriptive statistics of the dataset.

The total chlorophyll content varies widely, in a range higher than that reported in other studies [36]. The discrepancy between the mean and the median values indicates that some extreme samples are present in the dataset. In particular, four VOOs had a total chlorophyll amount over 100 mg/kg, while six were under 10 mg/kg. Chlorophyll content also depends on the maturity degree among other factors [37] and can have a relevant role in oil stability, acting an antioxidant in dark but as a pro-oxidant under light storage [13]. Carotenoids are another important class of VOOs natural minor compounds and antioxidants [13]. The range found in the VOOs studied was similar to that found in other reports [36] without any extreme sample. The total tocopherol content varied from about 100 mg/kg to just under 500 mg/kg, showing a wide range between the oils. The content was similar to, or even higher than, those found in other studies [25,38]. The alfa isoform represented the most abundant fraction in all cases, ranging from about 94% of total tocopherols to almost 100%. This is a relevant aspect to consider for possible valorization of these accessions. Indeed, alfa-tocopherol plays a dual role. From a nutritional perspective, it has the highest vitamin E activity of all tocochromanols [39] and some varieties (e.g., Unknown 3, Oleaster, Pasola, Pinziata, Rosciola Gentile, Silletta 2, Koroneki type), which have remarkable amount of total tocopherols represented by 98–99% of alfa-tocopherol, show potentiality for specific production, such as functional VOOs [40]. However, on the other hand, it is not considered the isoform with the highest antioxidant activity [12,13]. A correlation analysis revealed only a weak linear correlation between chlorophyll and carotenoid, while it was almost absent between the total tocopherols and the pigments. Overall, the results show how the studied accessions can be the source of different characters possibly useful to differentiate virgin olive oils [41].

## 4. Conclusions

Many reports over time have indicated that genuine VOOs can have purity characteristics out of the limits set by current regulations. The results of this work confirmed that this happens even in VOOs extracted from minor accessions of the Apulian territory (at least 1 parameter out of specification for 33 out of 48 samples). The issue should be carefully taken into account, also considering the possible effect due to the climate changes. The purity features of the samples allow them to be separated into 5 clusters, with different characteristics. According to the classical separation into high-oleic/low-palmitic–linoleic oils and vice versa, samples belonging to Cluster #3 (Ac’Lin, Cazznedd, Unknown 3, Oleaster, Parri, Peppino Leo, Piccolina, Pizzutella, Provenzale 2, Rosciola Gentile, Sannicandrese, Sant’Agostino, Unknown 1, Unknown 5, Silletta Nisi, Silletta 2, Termite di Bitetto, Koroneki type, Tonda Dolce) are low-oleic/high-palmitic–linoleic VOOs. On the other hand, samples belonging to Clusters #4 and #5 (Canua, Fragolino, Grappa, Unknown 4, Leucocarpa, Marinese, Rosciola, Rosciolone, Rumanella, S. Giovanni, Unknown 2, Tunnella, Uva, Bella di Spagna, Canua 2, Dolce di Massafra, Dritta Accadia, Mercurio, Nolca, Ogliarola di Biccari, Pendolino type 1, Pendolino type 2, Pinziata, Provenzale, Ravece) can be recognized as high-oleic/low-palmitic–linoleic oils. The San Benedetto variety stands alone for its peculiar characteristics, which deserve further study. Finally, the few samples from Cluster #1 (Dolce, Matarrese, Pasola) present intermediate features. The knowledge about purity characteristics of VOOs from minor varieties is mandatory from the perspective of targeted valorisation strategies as well as for a critical discussion about the current legislation limits the light of climate change. The samples also presented a wide range of pigments and tocopherol contents, highlighting peculiar characteristics. Several accessions produced a VOO with remarkable amounts of tocopherols (>350 mg/kg: Unknown 3, Nolca, Oleaster, Parri, Pasola, Pendolino type 2, Pinziata, Rosciola Gentile, SanBenedetto, Silletta 2, Koroneki type), an interesting trait for possible exploitation as functional oils. This study represents a contribution toward a comprehensive characterization of these accessions aiming at their possible valorisation. Nonetheless, it has been not possible highlighting the complex effect that genotype, agronomical practices, geographical origin, climatic conditions can have on those characteristics. Further studies should be designed to shed light on this matter.

## Figures and Tables

**Table 1:** Fatty acid composition (area percentage) and descriptive statistics of the dataset (n = 48).

Sample	C14:0	C16:0	C16:1	C17:0	C17:1	C18:0	C18:1	C18:2	C18:3	C20:0	C20:1	C22:0	C24:0
Ac’Lin	**0.05**	15.27	1.88	0.04	0.09	2.12	65.28	13.98	0.24	0.55	0.40	0.05	0.04
Bella di Spagna	0.00	16.53	2.09	0.00	0.10	2.52	70.77	6.63	0.29	**0.61**	0.36	0.11	0.00
Canua 1	0.03	11.61	1.05	0.02	0.02	1.68	80.16	4.14	0.33	0.55	0.32	0.11	0.00
Canua 2	0.00	14.39	1.65	0.00	0.00	2.48	71.06	9.11	0.35	0.52	0.28	0.09	0.07
Cazznedd	0.01	14.00	2.12	0.03	0.06	1.74	62.93	17.95	0.27	0.53	0.28	0.06	0.02
Dolce di Massafra	0.01	15.57	1.36	0.13	0.11	1.71	71.78	7.98	0.23	**0.68**	0.37	0.06	0.03
Dolce	0.00	11.10	0.80	0.20	0.20	2.20	74.00	10.30	0.30	0.40	0.50	0.10	0.00
Dritta Accadia	0.02	13.10	0.34	0.02	0.10	2.10	77.01	6.05	0.34	0.50	0.29	0.08	0.04
Fragolino	0.00	14.66	1.97	0.00	0.09	1.72	68.58	11.82	0.25	0.52	0.29	0.10	0.00
Grappa	0.01	11.54	1.37	0.03	0.08	1.66	80.98	3.29	0.27	0.35	0.34	0.07	0.03
Unknown 3	0.02	16.28	2.25	0.03	0.08	1.79	70.32	8.00	0.16	**0.63**	0.41	0.05	0.00
Unknown 4	0.00	13.20	1.00	0.00	0.10	1.60	69.30	13.40	0.30	**0.90**	0.20	0.00	0.00
Leucocarpa	0.00	15.75	2.22	0.15	0.11	1.66	72.27	6.79	0.17	0.56	0.23	0.10	0.00
Marinese	0.03	14.92	1.40	0.03	0.10	1.50	71.83	8.83	0.29	**0.61**	0.33	0.09	0.03
Matarrese	0.00	14.29	1.30	0.15	0.16	1.73	71.59	9.16	0.33	**0.64**	0.45	0.13	0.06
Mercurio	0.00	14.44	1.80	0.11	0.05	2.48	70.82	9.15	0.35	0.45	0.25	0.08	0.02
Nolca	0.00	12.72	1.22	0.10	0.07	2.62	71.88	9.94	0.29	**0.70**	0.39	0.06	0.00
Ogliarola di Biccari	0.00	14.82	1.30	0.07	0.02	2.43	70.87	8.91	0.35	0.60	0.31	**0.31**	0.00
Oleaster	**0.06**	19.59	2.03	0.07	0.09	2.06	60.77	13.63	0.27	**0.97**	0.33	0.08	0.05
Parri	0.00	16.17	2.56	0.16	0.12	1.79	66.43	11.56	0.29	0.58	0.28	0.06	0.00
Pasola	0.00	10.28	0.78	0.25	0.20	2.72	70.41	13.90	0.28	**0.61**	0.45	0.05	0.08
Pendolino type 1	0.02	14.10	1.25	0.06	0.09	2.39	71.20	9.48	0.37	0.57	0.23	0.14	0.10
Pendolino type 2	0.00	12.74	0.74	0.07	0.05	3.27	72.84	8.52	0.50	**0.74**	0.41	0.09	0.04
Peppino Leo	0.00	19.34	3.09	0.05	0.14	1.43	65.10	9.85	0.20	0.43	0.30	0.07	0.00
Piccolina	0.02	16.01	2.28	0.10	0.10	1.37	69.16	9.92	0.22	0.52	0.27	0.03	0.02
Pinziata	0.00	10.30	0.65	0.04	0.07	2.15	78.89	6.60	0.26	**0.62**	0.25	0.14	0.02
Pizzutella	0.02	16.01	2.28	0.10	0.10	1.37	69.16	9.92	0.22	0.52	0.27	0.03	0.02
Provenzale	0.01	13.26	0.98	0.05	0.09	3.05	72.54	8.44	0.54	0.53	0.31	0.14	0.05
Provenzale 2	0.00	15.63	1.22	0.03	0.00	1.64	65.78	14.11	0.21	**0.80**	0.40	0.16	0.00
Ravece	0.00	14.00	1.10	0.07	0.09	2.82	71.90	8.62	0.34	**0.71**	0.28	0.07	0.00
Rosciola	**0.05**	15.10	1.80	0.04	0.12	1.49	73.11	6.94	0.24	0.58	0.36	0.08	0.10
Rosciola Gentile	0.00	17.74	2.15	0.06	0.06	1.61	69.77	7.23	0.24	**0.63**	0.40	0.07	0.07
Rosciolone	0.02	13.83	1.91	0.06	0.04	1.68	74.49	6.88	0.23	0.43	0.36	0.06	0.01
Rumanella	0.00	13.59	0.83	0.05	0.08	1.72	73.60	8.93	0.26	0.44	0.37	0.09	0.05
S. Giovanni	**0.09**	14.55	0.72	0.19	0.11	1.49	73.38	8.34	0.16	**0.64**	0.33	0.00	0.00
SanBenedetto	0.00	14.73	0.93	0.15	0.24	1.38	70.19	11.22	0.66	0.35	0.06	0.08	0.00
Sannicandrese	0.00	16.42	2.94	0.19	0.06	1.52	64.21	13.64	0.14	0.44	0.34	0.07	0.02
Sant’Agostino	0.03	13.95	1.63	0.16	0.11	1.65	67.56	13.44	0.29	**0.76**	0.36	0.07	0.00
Unknown 2	0.03	15.42	1.53	0.06	0.04	1.55	67.75	12.25	0.24	**0.63**	0.40	0.07	0.03
Unknown 1	**0.04**	16.35	3.09	0.00	0.09	1.33	66.02	12.22	0.00	0.24	0.24	**0.31**	0.08
Unknown 5	0.00	17.53	1.60	0.04	0.13	1.61	63.77	13.93	0.36	0.52	0.36	0.11	0.05
Silletta Nisi	0.00	14.79	1.76	0.07	0.12	1.78	70.09	10.34	0.34	0.19	0.32	0.12	0.08
Silletta 2	**0.04**	16.37	1.46	0.16	0.12	1.69	69.29	9.55	0.25	0.58	0.39	0.11	0.00
Termite di Bitetto	0.03	16.13	**3.60**	0.05	0.06	1.91	66.92	9.98	0.24	**0.65**	0.34	0.06	0.04
Koroneki type	0.00	18.27	2.17	0.01	0.05	2.34	61.78	13.86	0.32	**0.82**	0.26	0.08	0.04
Tonda Dolce	0.00	14.90	2.06	0.06	0.08	1.52	70.92	9.44	0.20	0.28	0.32	0.11	0.10
Tunnella	0.01	15.60	1.11	0.05	0.07	1.76	70.60	9.32	0.30	**0.71**	0.36	0.08	0.03
Uva	0.00	14.16	1.86	0.07	0.03	1.85	71.26	9.54	0.17	0.58	0.41	0.08	0.00
Min	0.00	10.28	0.34	0.00	0.00	1.33	60.77	3.29	0.00	0.19	0.06	0.00	0.00
Max	0.09	19.59	3.60	0.25	0.24	3.27	80.98	17.95	0.66	0.97	0.50	0.31	0.10
Range	0.09	9.31	3.26	0.25	0.24	1.94	20.21	14.66	0.66	0.78	0.44	0.31	0.10
Mean	0.01	14.81	1.65	0.08	0.09	1.91	70.22	9.94	0.28	0.57	0.33	0.09	0.03
Median	0.00	14.81	1.62	0.06	0.09	1.73	70.69	9.51	0.27	0.58	0.33	0.08	0.02

In bold are values over the limit set in [2].

**Table 2:** Sterols composition (area percentage), total sterols (mg/kg), and descriptive statistics of the dataset (n = 48).

Sample	Cholesterol	Brassicasterol	Campesterol	Stigmasterol	Apparent Sitosterol	Delta-7-Stigmastenol	Total Sterols
Ac’Lin	0.2	0.0	2.7	1.6	**92.8**	0.2	1979
Bella di Spagna	0.0	0.0	3.2	0.7	93.4	0.2	1011
Canua 1	0.0	0.0	3.6	1.9	**91.4**	0.2	1857
Canua 2	0.1	0.0	3.2	1.2	93.7	0.3	1518
Cazznedd	0.1	**0.2**	2.8	1.6	**92.9**	0.3	1579
Dolce di Massafra	0.3	0.0	1.9	1.5	94.8	0.3	1390
Dolce	0.4	0.0	2.8	0.7	93.5	0.4	1717
Dritta Accadia	0.2	0.0	2.8	0.4	94.0	0.3	1288
Fragolino	0.2	0.0	3.5	1.3	93.2	0.4	2366
Grappa	0.1	0.0	2.7	2.3	**91.5**	0.5	1180
Unknown 3	0.3	0.0	2.2	1.3	93.6	0.1	1742
Unknown 4	0.3	0.0	2.4	2.2	**92.9**	0.4	2102
Leucocarpa	0.4	0.0	2.9	1.1	93.5	0.5	2403
Marinese	0.0	0.0	2.5	1.9	**92.5**	0.5	1664
Matarrese	0.2	**0.2**	3.1	0.8	93.3	0.4	1094
Mercurio	0.4	0.0	1.3	0.7	93.5	0.4	1647
Nolca	0.2	0.0	3.1	0.7	93.5	0.3	1203
Ogliarola di Biccari	0.0	0.0	2.4	1.7	93.1	0.1	1410
Oleaster	0.2	0.0	2.2	1.2	93.5	0.2	1758
Parri	0.0	0.0	2.4	0.7	93.3	0.4	1567
Pasola	0.0	0.0	2.5	1.9	93.0	0.5	1643
Pendolino type 1	0.2	0.0	2.5	1.2	93.2	0.3	2020
Pendolino type 2	0.3	0.0	3.0	0.8	93.5	0.2	2783
Peppino Leo	0.1	0.0	2.8	0.1	93.4	0.4	1601
Piccolina	0.2	0.0	2.4	1.3	93.4	0.2	1978
Pinziata	0.4	0.0	2.0	1.3	93.1	0.3	2073
Pizzutella	0.0	0.0	2.2	0.8	93.1	0.3	1738
Provenzale	0.2	0.0	2.7	0.9	94.0	0.3	1626
Provenzale 2	0.2	0.0	1.9	1.8	94.2	0.2	1978
Ravece	0.0	**0.1**	1.7	1.0	93.9	0.4	1941
Rosciola	0.2	0.0	2.8	0.7	93.9	0.4	1423
Rosciola Gentile	0.0	0.0	1.7	0.8	95.1	0.5	1752
Rosciolone	0.2	**0.2**	2.3	0.9	93.0	0.2	2164
Rumanella	0.2	0.0	3.3	2.1	**90.5**	**0.6**	2350
S. Giovanni	0.1	0.1	2.6	0.8	93.9	0.5	1490
SanBenedetto	0.1	0.0	2.6	**3.5**	**90.4**	0.4	1706
Sannicandrese	0.3	0.0	2.7	2.0	93.1	0.4	1234
Sant’Agostino	0.0	0.1	3.0	1.6	93.0	0.3	1631
Unknown 2	0.0	0.0	2.6	1.3	93.3	**0.6**	2243
Unknown 1	0.1	**0.2**	2.7	2.1	93.2	0.2	1614
Unknown 5	0.2	0.0	2.2	2.4	93.4	0.2	1325
Silletta Nisi	0.0	**0.3**	2.8	1.2	93.3	0.2	2215
Silletta 2	0.0	0.0	2.1	0.8	93.4	0.3	2119
Termite di Bitetto	0.0	0.0	2.3	1.1	93.1	0.5	1503
Koroneki type	0.2	0.0	2.4	1.7	93.1	0.4	1947
Tonda Dolce	0.2	0.0	2.0	0.3	94.2	0.4	1953
Tunnella	0.1	0.0	2.5	1.5	93.4	0.3	1531
Uva	0.2	0.0	3.1	**3.5**	**91.6**	0.3	2677
Min	0.0	0.0	1.3	0.1	90.4	0.1	1011
Max	0.4	0.3	3.6	3.5	95.1	0.6	2783
Range	0.4	0.3	2.3	3.4	4.7	0.5	1772
Mean	0.2	0.0	2.6	1.4	93.2	0.3	1765
Median	0.2	0.0	2.6	1.2	93.3	0.3	1711

In bold are values over the limit set in [2].

**Table 3:** Identified clusters, related olive accessions, and average characteristics of each cluster.

Cluster #	N	Accession			
1	3	Dolce, Matarrese, Pasola			
2	1	San Benedetto			
3	19	Ac’Lin, Cazznedd, Unknown 3, Oleaster, Parri, Peppino Leo, Piccolina, Pizzutella, Provenzale 2, Rosciola Gentile, Sannicandrese, Sant’Agostino, Unknown 1, Unknown 5, Silletta Nisi, Silletta 2, Termite di Bitetto, Koroneki type, Tonda Dolce			
4	13	Canua 1, Fragolino, Grappa, Unknown 4, Leucocarpa, Marinese, Rosciola, Rosciolone, Rumanella, S. Giovanni, Unknown 2, Tunnella, Uva			
5	12	Bella di Spagna, Canua 2, Dolce di Massafra, Dritta Accadia, Mercurio, Nolca, Ogliarola di Biccari, Pendolino type 1, Pendolino type 2, Pinziata, Provenzale, Ravece			
			**Cluster #**		
**Variable**	**1**	**2**	**3**	**4**	**5**
C14:0	0.00	0.00	0.02	0.02	0.01
C16:0	11.89	14.73	16.36	14.15	13.83
C16:1	0.96	0.93	2.22	1.44	1.21
C17:0	0.20	0.15	0.07	0.06	0.06
C17:1	0.19	0.24	0.09	0.08	0.07
C18:0	2.22	1.38	1.70	1.64	2.50
C18:1	72.00	70.19	66.59	72.87	72.63
C18:2	11.12	11.22	11.71	8.50	8.29
C18:3	0.30	0.66	0.23	0.25	0.35
C20:0	0.55	0.35	0.56	0.58	0.60
C20:1	0.47	0.06	0.33	0.33	0.31
C22:0	0.09	0.08	0.09	0.07	0.11
C24:0	0.05	0.00	0.03	0.02	0.03
Cholesterol	0.2	0.1	0.1	0.1	0.2
Brassicasterol	0.1	0.0	0.0	0.0	0.0
Campesterol	2.8	2.6	2.4	2.8	2.5
Stigmasterol	1.1	3.5	1.3	1.6	1.0
Apparent sitosterol	93.3	90.4	93.4	92.7	93.6
Delta-7-stigmastenol	0.4	0.4	0.3	0.4	0.3
Total sterols	1484	1706	1748	1958	1659

**Table 4:** Pigments and tocopherols content and descriptive statistics of the dataset (n = 48).

Sample	Chlorophylls (mg/kg)	Carotenoids (mg/kg)	Tocopherols (mg/kg)
Ac’Lin	2.06	6.49	184.69
Bella di Spagna	30.13	20.64	197.13
Canua 1	27.44	12.29	191.70
Canua 2	25.96	8.41	231.92
Cazznedd	7.90	8.49	210.23
Dolce di Massafra	13.59	16.53	156.92
Dolce	12.91	7.59	340.44
Dritta Accadia	123.96	10.09	217.73
Fragolino	13.57	8.72	186.71
Grappa	4.35	4.15	214.70
Unknown 3	82.94	14.81	375.20
Unknown 4	23.79	5.08	207.92
Leucocarpa	65.41	4.26	344.97
Marinese	54.94	12.45	221.21
Matarrese	111.72	22.71	187.32
Mercurio	14.42	5.48	149.50
Nolca	17.78	12.56	400.92
Ogliarola di Biccari	68.89	8.49	224.41
Oleaster	129.84	39.74	489.45
Parri	20.59	11.30	403.55
Pasola	20.84	11.10	354.74
Pendolino type 1	32.34	13.13	272.31
Pendolino type 2	48.09	16.02	362.85
Peppino Leo	12.32	4.89	130.26
Piccolina	66.65	6.28	221.17
Pinziata	35.33	21.88	473.42
Pizzutella	8.45	4.26	317.89
Provenzale	35.37	8.22	233.99
Provenzale 2	80.47	7.66	245.47
Ravece	21.48	10.50	123.15
Rosciola	20.38	11.68	292.65
Rosciola Gentile	23.89	10.52	458.52
Rosciolone	35.08	6.70	308.52
Rumanella	38.98	11.69	260.27
S. Giovanni	23.34	5.03	240.43
SanBenedetto	8.32	7.08	482.45
Sannicandrese	23.27	1.46	299.25
Sant’Agostino	13.39	7.90	231.54
Unknown 2	34.49	8.19	242.53
Unknown 1	30.09	6.51	286.35
Unknown 5	33.18	4.33	270.84
Silletta Nisi	111.22	11.03	312.74
Silletta 2	22.90	10.37	390.68
Termite di Bitetto	13.50	8.01	134.40
Koroneki type	51.24	8.25	409.87
Tonda Dolce	7.00	3.52	304.90
Tunnella	50.80	10.96	262.24
Uva	14.43	6.08	227.20
Min	2.06	1.46	123.15
Max	129.84	39.74	489.45
Range	127.77	38.27	366.31
Mean	36.85	10.07	276.82
Median	24.93	8.45	252.87

## Data Availability

The original contributions presented in this study are included in the article/Supplementary Materials. Further inquiries can be directed to the corresponding author.

## Supplementary Materials

The following supporting information can be downloaded at: https://www.mdpi.com/article/10.3390/foods14060964/s1, Table S1: Collection province of the olive accessions considered.
