# Supplementary material for: An Overview of the Purity Characteristics, Pigments, and Tocopherol Contents of 48 Virgin Olive Oils from Apulian Minor Olive Accessions
Source: Foods. 2025 Mar 12;14(6):964. doi: 10.3390/foods14060964 (PMC11941103; doi:10.3390/foods14060964)
Supplement: Supplementary file 1 [file foods-14-00964-s001.zip › foods-3481470-supplementary.pdf]

Table S1. Collection province of the olive accessions considered.

Name	Province
Ac'Lin	n.a.
Bella di Spagna	n.a.
Canua	Bari
Canua	Bari
Cazznedd	n.a.
Dolce di Massafra	Taranto
Dolce	n.a.
Dritta Accadia	Foggia
Fragolino	Foggia
Grappa	Brindisi
Unknown 3	Bari
Unknown 4	Bari
Leucocarpa	Foggia
Marinese	Foggia
Matarrese	Bari
Mercurio	n.a.
Nolca	Bari
Ogliarola di Biccari	Foggia
Oleaster	Foggia
Parri	n.a.
Pasola	Brindisi
Pendolino type 1	Foggia
Pendolino type 2	Foggia
Peppino Leo	Bari
Piccolina	Bari
Pinziata	Foggia
Pizzutella	Bari
Provenzale	Foggia
Provenzale 2	Foggia
Ravece	Foggia
Rosciola	Foggia
Rosciola Gentile	Foggia
Rosciolone	Foggia
Rumanella	Foggia
S. Giovanni	Bari
SanBenedetto	n.a.
Sannicandrese	Bari
Sant'Agostino	Foggia
Unknown 2	n.a.

Unknown 1	n.a.
Unknown 5	Foggia
Silletta Nisi	Brindisi
Silletta 2	Bari
Termitedi Bitetto	Bari
Koroneki type	Foggia
Tonda Dolce	Foggia
Tunnella	Foggia
Uva	n.a.

n.a., not available
